# Draft Genome Sequence of *Chlorobium* sp. Strain N1, a Marine Fe(II)-Oxidizing Green Sulfur Bacterium

**DOI:** 10.1128/MRA.00080-19

**Published:** 2019-05-02

**Authors:** Casey Bryce, Nia Blackwell, Daniel Straub, Sara Kleindienst, Andreas Kappler

**Affiliations:** a Geomicrobiology Group, Center for Applied Geoscience, University of Tübingen, Tübingen, Germany; b Microbial Ecology Group, Center for Applied Geoscience, University of Tübingen, Tübingen, Germany; c Quantitative Biology Center (QBiC), University of Tübingen, Tübingen, Germany; Indiana University, Bloomington

## Abstract

Here, we present the draft genome sequence of the halotolerant photoferrotroph Chlorobium sp. strain N1. This draft genome provides insights into the genomic potential of the only marine Fe(II)-oxidizing green sulfur bacterium (GSB) available in culture and expands our views on the metabolic capabilities of Fe(II)-oxidizing GSB more generally.

## ANNOUNCEMENT

Anoxygenic photoautotrophic Fe(II)-oxidizing bacteria, so-called “photoferrotrophs,” were key drivers in the marine iron cycle prior to the rise of oxygen (1). Numerous photoferrotrophs have been isolated (reviewed in reference 2) that are either purple sulfur/nonsulfur bacteria or green sulfur bacteria (GSB), but most are from freshwater environments. Here, we present the genome sequence of the halotolerant GSB Chlorobium sp. strain N1, which is the only Fe(II)-oxidizing GSB obtained from a marine environment (3) and thus provides a unique opportunity to study Fe(II)-oxidizing GSB at salinities comparable to those of the ancient oceans.

Chlorobium sp. strain N1 was obtained from sediments of Norsminde Fjord in Aarhus Bay, Denmark, and was maintained in artificial seawater medium with 10 mM FeCl_2_ (the physiology of Chlorobium sp. N1 is described in reference 3). Genomic DNA was extracted using the PowerSoil DNA extraction kit, according to the manufacturer’s instructions, and stored at −80°C. Three hundred nanograms of high-molecular-weight DNA was sheared to a target fragment size of 550 bp using a Covaris S2 focused ultrasonicator, and genomic sequencing libraries were prepared using the NEBNext Ultra II DNA library preparation for Illumina sequencing kit (New England BioLabs), according to the manufacturer’s protocol. Sequencing was conducted by IMGM Laboratories GmbH (Munich, Germany) with the Illumina MiSeq kit version 2. A total of 3,003,794 read pairs with a 250-bp read length were produced. Reads were trimmed and adapters removed using Trimmomatic version 0.36 (4), with the following parameters: ILLUMINACLIP:TruSeq3-PE-2.fa:2:30:10:1:true, LEADING:3, TRAILING:3, SLIDINGWINDOW:4:15, and MINLEN:50. The quality was checked with FastQC version 0.11.5 before and after trimming, using the default parameters (5).

PhiX contamination was tested with Bowtie version 2.3.3 by aligning trimmed paired-end reads to the PhiX genome (Illumina iGenome). Forty-five percent of the paired-end reads were successfully merged with FLASH version 1.2.11 (6). Merged and unmerged paired-end reads were subsequently assembled with SPAdes version 3.11.0 (7). Genome contamination of 66.20% was estimated by CheckM version 1.0.11 (8) with Prodigal version 2.6.2 (9), HMMER version 3.1b2 (http://hmmer.org/), and pplacer version 1.1.alpha19 (10) based on the reduced reference genome tree using the current CheckM database. The GC content peaked at around 35% and 65%, with similar peak heights, making the presence of one or more other genomes likely. *In silico* genome purification with MaxBin version 2.2.4 (11) extracted 36 contigs with an *N*_50_ value of 265,932 bp. Using these purified contigs, CheckM calculated a genome completeness for Chlorobium sp. strain N1 of 98.91% and found no contamination. The contigs were uploaded to the Integrated Microbial Genomes Expert Review tool (https://img.jgi.doe.gov/cgi-bin/submit/main.cgi) for gene predictions and functional annotations.

The Chlorobium sp. strain N1 genome is 2,373,849 Mb, with 2,287 open reading frames (ORFs), of which 2,217 are protein-coding genes. One full-length 16S rRNA gene sequence was found, as well as a 16S rRNA gene fragment. At the 16S rRNA gene level, this strain is 99% similar to Chlorobium luteolum DSM 273, which has not yet been shown to oxidize Fe(II), but it has only 80% similarity at the genome level. This strain is 72% similar at the genome level to freshwater Chlorobium ferrooxidans, the first Fe(II)-oxidizing GSB identified.

This draft genome contains genes for the type I photosynthetic reaction center, CO_2_ fixation via the reverse tricarboxylic acid (TCA) cycle, and sulfur metabolism pathways common to the *Chlorobi*, as well as for multiple fermentation pathways. A homolog to the Cyc2 iron oxidase of Acidithiobacillus ferrooxidans is also present. This draft genome not only provides the first insights into the genomic potential of the only marine Fe(II)-oxidizing GSB obtained in culture, but it also expands our views on the metabolic capabilities of the Fe(II)-oxidizing GSB more generally.

### Data availability.

This genome can be accessed at http://img.jgi.doe.gov, and associated metadata can be found in the GOLD database at https://gold.jgi.doe.gov/ (GOLD analysis project identifier [ID] Ga0226664). This whole-genome shotgun project has been deposited at DDBJ/ENA/GenBank under the accession number PRJEB30018, including the assembly (accession number SJPA00000000). The raw data have been deposited in the Sequence Read Archive under accession number ERS2924416.
